# The Human Lung Adenocarcinoma Cell Line EKVX Produces an Infectious Xenotropic Murine Leukemia Virus

**DOI:** 10.3390/v3122442

**Published:** 2011-12-19

**Authors:** Joan L. Cmarik, Jami A. Troxler, Charlotte A. Hanson, Xiang Zhang, Sandra K. Ruscetti

**Affiliations:** Laboratory of Cancer Prevention, National Cancer Institute-Frederick, Frederick, MD 21702, USA; Email: ruscetts@mail.nih.gov (S.K.R.)

**Keywords:** EKVX, NCI-60, human cell line, xenotropic murine leukemia virus, gammaretrovirus

## Abstract

The cell lines of the NCI-60 panel represent different cancer types and have been widely utilized for drug screening and molecular target identification. Screening these cell lines for envelope proteins or gene sequences related to xenotropic murine leukemia viruses (X-MLVs) revealed that one cell line, EKVX, was a candidate for production of an infectious gammaretrovirus. The presence of a retrovirus infectious to human cells was confirmed by the cell-free transmission of infection to the human prostate cancer cell line LNCaP. Amplification and sequencing of additional proviral sequences from EKVX confirmed a high degree of similarity to X-MLV. The cell line EKVX was established following passage of the original tumor cells through nude mice, providing a possible source of the X-MLV found in the EKVX cells.

## 1. Introduction

Continuous cell lines established from human cancer specimens are critical to cancer research. Biosafety guidelines have been established for handling these cell lines because of the potential presence of infectious agents. The NCI-60 panel comprises cell lines derived from human tumors from various organs (Table 1). The panel was developed by the Developmental Therapeutics Program (DTP) of the National Cancer Institute (NCI) as an *in vitro* drug screening tool [1], and at the time of its establishment, all cell lines were tested for known pathogens [2]. The NCI-60 panel is currently used to screen up to 3000 synthetic compounds or natural products per year for potential anticancer activity and is also frequently used for molecular target identification (dtp.nci.nih.gov/branches/btb/ivclsp.html). The DTP has supplied the cell lines of the panel to a large number of other research laboratories. Because of their widespread use, the discovery of xenotropic murine leukemia virus-related virus (XMRV) prompted us to screen the NCI-60 cell lines for previously undetected infectious retroviruses. 

**Table 1:** Derivation of the cell lines of the NCI-60 panel and results of virus screening.

Cell line name	Tumor type	Immuno-blotting for Env	PCR for *env*^a^	PCR for *gag*^a^	Passaged through mice^b^	Reference
BT-549	Breast	-	-	-	N^c^	
Hs-578T	Breast	-	-	-	N	[3]
MCF-7	Breast	-	-	-	N	[4]
MDA-MB-231	Breast	-	-	-	N	[5]
T-47D	Breast	-	-	-	N	[6]
MDA-MB-468	Breast	-	-	-	N	[7]
SF-268	CNS	-	-	-	N	[8]
SF-295	CNS	-	-	-	N	[8]
SF-539	CNS	-	-	-	N	[9]
SNB-19^d^	CNS	-	-	-	N	[10,11,12]
SNB-75	CNS	-	-	-	N	[10,11]
U251^d^	CNS	-	-	-	N	[12,13]
COLO-205	Colon	-	-	-	N	[14]
HCC-2998	Colon	-	-	-	Y^e^	
HCT-116	Colon	-	-	-	N	[15]
HCT-15	Colon	-	-	-	N	[16]
HT29	Colon	-	-	-	N	[17]
KM12^f^	Colon	-	-	-	N	[18]
SW-620	Colon	-	-	-	N	[19]
CCRF-CEM	Leukemia	-	-	-	N	[20]
HL-60	Leukemia	-	-	-	N	[21]
K562	Leukemia	-	-	-	N	[22]
MOLT-4	Leukemia	-	-	-	N	[23]
RPMI-8226	Leukemia	-	-	-	N	[24]
SR	Leukemia	-	-	-	N	[25]
LOX IMVI	Melanoma	-	-	-	Y	[26,27]
M14^g^	Melanoma	-	-	-	N	[28,29]
MALME-3M	Melanoma	-	-	-	N	[17]
MDA-MB-435^g^	Melanoma	-	-	-	N	[7,29,30,31]
SK-MEL-2	Melanoma	-	-	-	N	[17]
SK-MEL-28	Melanoma	-	-	-	N	[32]
SK-MEL-5	Melanoma	-	-	-	N	[32]
UACC-257	Melanoma	-	-	-	N^e^	
UACC-62	Melanoma	-	-	-	N^e^	
A549 ATCC	Non-Small Cell Lung	-	-	-	N	[33]
EKVX	Non-Small Cell Lung	+	+	+	Y	[26]
HOP-62	Non-Small Cell Lung	-	-	-	Y^e^	[34,35]
HOP-92	Non-Small Cell Lung	-	-	-	N^e^	[34,35]
NCI-H226	Non-Small Cell Lung	-	-	-	N	[36,37]
NCI-H23	Non-Small Cell Lung	-	-	-	N	[36,37]
NCI-H322M	Non-Small Cell Lung	-	-	-	N	[37,38,39]
NCI-H460	Non-Small Cell Lung	-	-	-	N	[36,37]
NCI-H522	Non-Small Cell Lung	-	-	-	N	[36,37]
IGR-OV1	Ovarian	-	-	-	N	[40]
NCI/ADR-RES^h^	Ovarian	-	-	-	N	[41,42,43]
OVCAR-3	Ovarian	-	-	-	N	[44]
OVCAR-4	Ovarian	-	-	-	N	[45]
OVCAR-5	Ovarian	-	-	-	N	[45]
OVCAR-8	Ovarian	-	-	-	N	[45,46]
SK-0V-3	Ovarian	-	-	-	N	[17]
DU145	Prostate	-	-	-	N	[47]
PC-3	Prostate	-	-	-	N	[48]
A498	Renal	-	-	-	N	[33]
ACHN	Renal	-	-	-	N	[49]
CAKI-1	Renal	-	-	-	N	[17]
RXF-393	Renal	-	-	-	Y	[50]
SN12C	Renal	-	-	-	N	[51]
TK-10	Renal	-	-	-	N	[52]
UO-31	Renal	-	-	-	U^i^	
786-0	Renal	-	-	-	N	[53]

^a^ “+”indicates a definitive band of the expected size was observed.

^b^ Y=Yes. N=No. U=Unknown; no information was available from literature references.

^c^ This cell line was obtained from ATCC. According to the ATCC description: “The BT-549 line was isolated in 1978 by W.G. Coutinho and E. Y. Lasfargues. Source tissue consisted of a papillary, invasive ductal tumor which had metastasized to 3 of 7 regional lymph nodes.” The establishment of other breast cancer cell lines by the same group has been described [54].

^d^ SNB-19 and U251 are derived from the same individual [12].

^e^ Michael Alley, NCI, personal communication.

^f^ The KM12 cell line is the same as KM12C in Morikawa *et al.* [18] (Michael Alley, NCI, personal communication).

^g^ Current samples of M14 and MDA-MB-435 are related [29]. MDA-MB-435 was originally isolated as a breast cancer cell line.

^h^ NCI/ADR-RES was originally described as a breast cancer cell line, but is related to the ovarian cancer cell line OVCAR-8 [41].

^i^ Highly unlikely to have been passaged through mice (W. Marston Linehan, NCI, personal communication).

XMRV is a type C gammaretrovirus first identified in human prostate tumors [55], although considerable controversy surrounds the detection of XMRV in human samples [56,57]. The prostate cancer cell line 22Rv1 produces infectious XMRV [58], but other prostate cancer cell lines, e.g., DU145 and LNCaP, do not [59]. Hue *et al.* [60] recently reported that 2.2% of a large number of cell lines tested by PCR were positive for xenotropic murine leukemia virus (X-MLV) *gag* sequence. We used immunoblotting and PCR assays capable of detecting XMRV and other X-MLVs to screen the NCI-60 panel of tumor cell lines. 

## 2. Results and Discussion

### 2.1. The Lung Cancer Cell Line EKVX Tested Positive by Both Immunoblotting for Viral Envelope Protein and by PCR for *gag* and *env* Viral Gene Sequences

**Figure 1:**
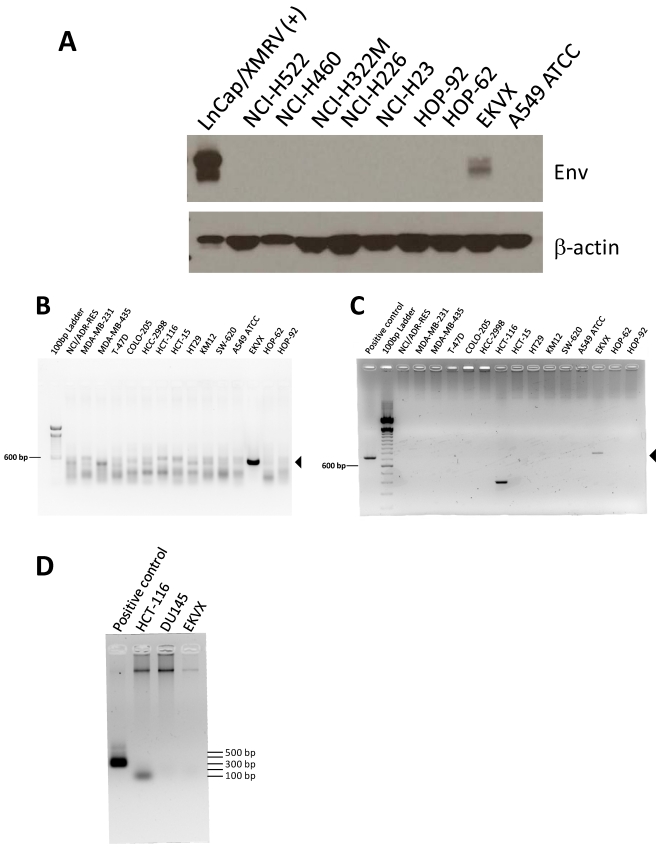
Detection of viral protein and DNA in the human lung adenocarcinoma cell line EKVX. Immunoblotting and PCR were carried out on all 60 cell lines of the panel; a subset are shown. Immunoblotting of total protein lysates from lung cancer cell lines for Env with monoclonal antibody 7C10 **(A)**. XMRV-infected LNCaP cell lysate [LNCaP/XMRV(+)] is included as a positive control. Env is present as both a precursor form (upper band) and a processed surface unit (lower band). Immunoblotting with β-actin antibody was used to confirm equal loading. Single-round PCR of genomic DNA for *env* **(B)** and *gag* **(C)** sequences. Template is genomic DNA from cell lines of the NCI-60 panel. Arrowheads indicate the expected fragment sizes: 533 bp for *env* and 731 bp for *gag*. Negative (no template) controls were run on separate gels and no products of the expected size were observed (data not shown).** (D)** EKVX and other representative cell lines, DU145 and HCT-116, were tested for mouse contamination by PCR for IAP using 600 ng genomic DNA as template. The positive control is genomic DNA from a mouse cell line diluted 1/10^5^ in human cell line genomic DNA.

Total protein lysates were prepared from all 60 cell lines. Viral envelope protein (Env, also known as gp70) was detected by immunoblotting with monoclonal antibody 7C10 [61], which was raised against the envelope protein of Friend spleen focus-forming virus and which cross-reacts with XMRV and other X-MLV envelope proteins. A positive signal was detected in the lysate of only 1 cell line, EKVX (Table 1 and Figure 1A), a lung adenocarcinoma– derived cell line [26]. As a second means of screening for virus, PCR for XMRV and X-MLV– related *gag* and *env* sequences was performed. In concordance with the immunoblotting results, only EKVX yielded prominent bands of the expected size with both the *env* and *gag* primers, 533 bp and 731 bp, respectively (Table 1 and Figures 1B and 1C). This result is consistent with Hue *et al.* [60], who detected MLV-like sequences in EKVX by PCR. To rule out the possibility that EKVX cultures or derived materials were contaminated with mouse cells or DNA containing endogenous X-MLVs that could give rise to the *env* and *gag* products, we used nested PCR as described by Ono *et al.* [62] to test for the presence of mouse mitochondrial DNA. Although we could reliably detect 1/10^6^ equivalents of mouse genomic DNA spiked into human genomic DNA, no mouse mitochondrial PCR products were observed with up to 250 ng of EKVX genomic DNA (data not shown). We were also unable to detect mouse DNA contamination using a single-round (45 cycles) PCR assay for intracisternal A particles (IAP) [63] on up to 600 ng of EKVX genomic DNA (Figure 1D). The mouse IAP products are ~200-300 bp; the ~100 bp band in the HCT-116 lane may be a primer dimer product, as it was observed frequently in no template controls in additional experiments. 

We observed a faint ladder of background bands in all cell lines with the *env* primers. Therefore, we tested a subset of first round *env* products with nested primers, and a band of the appropriate size was only detected with EKVX (data not shown). The first round background bands were not observed in two representative cell line DNA samples (HCT-116 and DU145) spiked with a small amount (1/10^6^) of genomic DNA containing the target DNA sequence (data not shown). Because no mouse DNA was detected in HCT-116 or DU145 genomic DNA using the IAP assay (Figure 1D), the background bands may have been the result of mispriming on human DNA sequences. A few cell lines gave rise to products of incorrect size with the *gag* primers (*e.g*., HCT-116 in Figure 1C), but no corresponding envelope PCR product was observed, and no product was detected using nested primers for *gag* (data not shown). 

Lung can serve as a point of entry for virus infection. A search of the GEO database [64] reveals numerous examples of expression of the X-MLV/XMRV receptor XPR1 [65] in human lung cells. Although potential contamination with mouse sequences was not ruled out, XMRV sequences were reported in human respiratory secretions [66], suggesting that lung could be a target for MLV-like viruses. However, all 8 of the other lung cancer cell lines in the NCI-60 panel and 4 additional lung cancer cell lines [NCI-H1666, NCI-H650, NCI-H2122, NCI-H358; obtained from M. Lerman, NCI (data not shown)] that we tested by immunoblotting were negative for MLV-like virus. 

### 2.2. EKVX Produces Virus Capable of Infecting Human Cells

**Figure 2:**
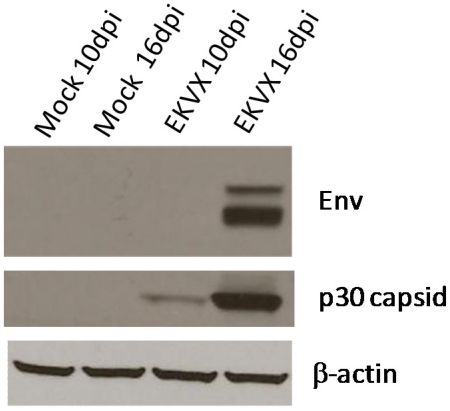
Transmission of infection by a cell-free EKVX supernatant. Subconfluent LNCaP cells were infected with filtered supernatant from EKVX cells (EKVX) or with cell culture medium as a negative control (Mock) in the presence of polybrene. Total protein lysates were prepared at 10 and 16 days post-infection (dpi). Lysates were subjected to electrophoresis and immunoblotting with monoclonal antibody 7C10 for Env and with goat anti-RLV p30 capsid. Immunoblotting with β-actin antibody was used to confirm equal loading.

Cell-free supernatants were prepared from cultures of EKVX cells. Reverse transcriptase activity was detected in the EKVX supernatant using manganese, but not magnesium (data not shown), indicative of a mammalian type C retrovirus reverse transcriptase [67]. The ability of the EKVX viral supernatant to infect and spread in human cells was assessed using the human prostate cancer cell line LNCaP. LNCaP cells pretreated with polybrene were incubated with cell-free EKVX supernatant or fresh media (negative control). Cells were subcultured and total protein lysates were prepared at 10 and 16 days post-infection. Analysis by immunoblotting with monoclonal antibody 7C10 and with goat anti-Rauscher MLV p30 indicated the presence of capsid and envelope viral proteins in the lysate (Figure 2), indicating the establishment and spread of infection from the EKVX supernatant. 

### 2.3. The EKVX Virus Is a Xenotropic Murine Leukemia Virus

**Figure 3:**
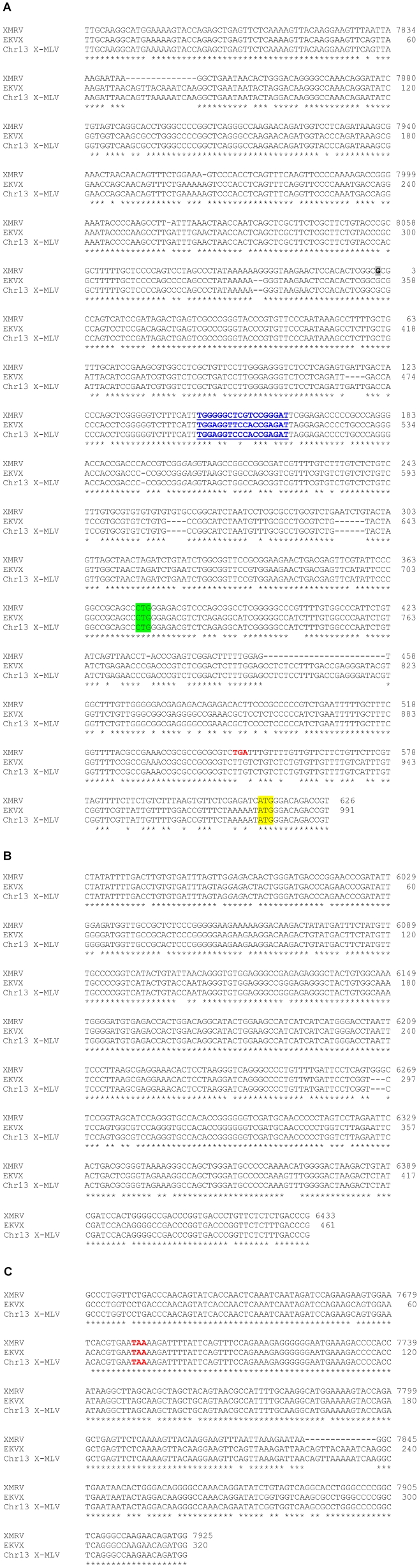
Nucleotide sequence from the LTR, *gag*, and *env* regions of the EKVX virus. The sequences of regions of the EKVX virus are aligned with XMRV (VP62; accession no. DQ399707) and an endogenous X-MLV provirus from chromosome 13 (Chr13 X-MLV) of a C57BL/6 mouse (accession no. CT030655, nt 54,685-63,371). U3 sequence from the 3′ end of XMRV VP62 sequence was appended to the 5′ end in the figure to generate the predicted provirus sequence. Numbering for XMRV is according to XMRV VP62, with nt 1 (at the U3/R transition) highlighted in gray. Numbering for EKVX indicates the nucleotide number for each fragment shown. Conserved nucleotides are indicated by *. **(A)** LTR-*gag* leader region, compiled from PCR fragments generated with primers DG-75 7762F/DG-75 287R and DG-75 7945F/DG-75 664R. The primer binding site is underlined and shown in blue. The upstream glyco-Gag start is highlighted in green, and the in-frame stop codon in XMRV is shown in red. The start codon for the Gag polyprotein is highlighted in yellow. **(B)** *env* region, generated with primers VP62 5922F / VP62 6454R. **(C)** *env*-LTR region, generated with primers DG-75 7607F/DG-75 7968R. The stop codon for the Env polyprotein is shown in red.

The nucleotide sequences of the *gag* and *env* PCR fragments amplified from EKVX genomic DNA were determined. Sequences of the EKVX *gag* and *env* fragments were more closely related to X-MLVs than to XMRV (98% vs. 87% identity for *gag*; 99% vs. 92% identity for *env*). Based on these results, primers were designed to amplify additional regions in the LTR, *gag*, and *env* based on the sequence of DG-75 X-MLV. The sequences of these PCR products were determined and are aligned with representative XMRV and X-MLV sequences in Figure 3. The LTR-*gag* region of the EKVX virus lacks several of the defining characteristics of XMRV while aligning with 99% identity with the X-MLV sequence; most notably, it lacks the 14 bp deletion found in U3 and the short additions and 24 bp deletion found in the *gag*-leader of XMRV (Figure 3A). The primer binding sites for the EKVX sequence and the X-MLV sequence shown in Figure 3A are complementary to Gln-tRNAs, whereas that for XMRV is complementary to Pro-tRNA [68]. Gln-tRNA binding sites have been reported for numerous endogenous MLVs [69,70,71]. Also of note within the *gag*-leader region, MLV sequences contain an alternative (CTG) start codon, upstream of the ATG start codon, that yields a glycosylated precursor (glyco-Gag) [72]. In contrast to XMRV, which has an in-frame stop codon that precludes glyco-Gag synthesis [55], there is no stop codon between the CTG and ATG *gag* start codons and they are in frame with each other, suggesting that the EKVX virus should produce glyco-Gag. The *env* region (Figure 3B) lacks the trinucleotide G insertion found in XMRV, and the *env*-LTR region (Figure 3C) is missing the same 14 bp deletion in U3 as noted in Figure 3A. Together, these sequence data confirm that the EKVX virus is more closely related to X-MLVs than to the reported sequences of XMRV. 

Xenotropic MLVs are so named because they can infect non-mouse species but cannot infect most laboratory strains of mice. X-MLV sequences are present as endogenous viruses in all laboratory and some house mouse strains [73,74,75,76,77], and endogenous viruses can be activated to produce infectious virus under certain conditions such as chemical or radiation exposure or immune induction [78,79,80,81,82]. Because EKVX was derived by passage through a nude mouse [26], the virus in the cell line may have been present in the original tumor or may have been acquired from nude mouse cells producing X-MLV. Several instances of human tumors and cell lines passed through nude mice having acquired viruses from the host have been reported [83,84,85,86,87,88,89]. However, not all cells passaged in mice acquire a virus. Indeed, four other cell lines of the NCI-60 panel are known to have been established after passage through mice (Table 1), and these tested negative for X-MLVs. The propensity for acquisition of a virus may depend on the strain of mouse, the means of immune suppression, the characteristics of the xenografted tumor, and whether the experimental protocol includes any additional factors (chemical exposure, radiation) that could promote the activation of an endogenous virus. Suzuki *et al.* [86] found that cells from 6/9 tumors transplanted into nude mice produced infectious murine type-C virus. Lusso *et al.* [85] found that cells recovered from all 6 human hematopoietic tumors studied that were transplanted into splenectomized, irradiated, and anti-asialo-GM1–treated nude mice acquired X-MLV infections. In other studies in which a variety of solid tumors were implanted as xenografts in nude mice or mice treated with mouse thymocyte antiserum, only 1/9 [87], 1/12 [84], and 2/11 [88] xenografts were associated with type C retroviruses. Intriguingly, Tralka *et al.* [88] reported IAP production in the human osteosarcoma cells that had acquired X-MLV infection. In a recent study, Zhang *et al.* [89] found evidence that 6/23 (mouse DNA-free) human cell lines established following passage through mice had been infected with X-MLV. Because of the possibility that the EKVX human lung cancer cell line acquired its X-MLV upon passage through nude mice, our study sheds no light on the controversy of whether X-MLV infections occur naturally in the human population. 

The identification of a human infectious retrovirus in a commonly handled cell line is of concern on multiple counts: 1) unknown risk of infection and health risks to persons exposed to the virus, 2) unanticipated influence of infection and expression of viral proteins on experimental results [85,86], and 3) risk of spread of infectious virus to other cultured cell lines [89]. Infection of cells with X-MLV significantly altered the interactions of HIV-1 with those cells compared to their uninfected counterparts [85]. With respect to the potential for unintended spread of the virus among cultures, two reported examples of X-MLV in human cell lines that had not been transplanted into mice appear to have resulted from vertical transmission of virus from another infected cell line [90,91]. Zhang *et al.* [89] found that non-xenograft cell lines maintained in a xenograft-free facility showed no evidence of MLV infection, whereas 17% of non-xenograft cell lines cultured in laboratories that also maintained xenograft cultures became infected with MLV. Our study indicates that the X-MLV present in the EKVX cell line has not spread to other cell lines of the NCI-60 panel maintained by the DTP. 

Our results confirm the findings of Sfanos et al. [92], who published the results of a similar study while this manuscript was in preparation for publication. They tested 58 of the 60 cell lines of the NCI-60 panel, and like us, found EKVX to be the only one of these cell lines expressing an X-MLV. An important difference between their study and ours is that the cell lines tested by Sfanos *et al*. were obtained from the NCI and then cultured in their own laboratory, whereas our initial screen was conducted on materials (DNA and cell pellets) directly supplied by the DTP, providing an additional level of confidence that the virus is present in the source cell line maintained by the DTP. Additionally, we utilized a different envelope antibody and different PCR primers, further strengthening the validity of the findings of both papers.

## 3. Experimental Section

### 3.1. Cell Line Materials and Cell Culture

Frozen cell pellets and genomic DNA were obtained from the DTP. EKVX cells were obtained from the DTP, and LNCaP cells were obtained from the laboratory of F. Ruscetti, NCI. Both cell lines were cultured in RPMI supplemented with penicillin/streptomycin and 10% fetal bovine serum. 

### 3.2. Immunoblotting

Total protein lysates were prepared as previously described [93], and 30 μg were subjected to electrophoresis on 4-12% Bis-Tris gels (Invitrogen, Carlsbad, CA) with MOPS buffer and transferred to nitrocellulose. Monoclonal antibody 7C10 [61], polyclonal goat antisera against Rauscher MLV p30 (NCI, Bethesda, MD), and β-actin antibody (Novus Biologicals, Littleton, CO) were used as primary antibodies. Detection was accomplished with horseradish peroxidase-labeled secondary antibodies and enhanced chemiluminescent substrate (Thermo Scientific, Rockford, IL). XMRV-infected LNCaP cell lysate, provided by F. Ruscetti, was used as a positive control. 

### 3.3. PCR

PCR for XMRV *gag* sequences was carried out on 75 ng of genomic DNA in a 25 μl reaction using primers 419F and 1154R and conditions as previously described [94] except the final concentration of each primer was 0.2 μM and the extension step was shortened to 45 s. PCR for XMRV *env* sequences was carried out similarly with primers 5922F (5′-GCTAATGCTACCTCCCTCCTGG) and 6454R (5′-GGCCCTACATTGAGGACCTGG) with an annealing temperature of 58°C. Hot-StartIt FideliTaq (USB, Cleveland, OH) was used for PCR. No template (water) controls and an XMRV-infected positive control (provided by F. Ruscetti) were included. In some cases, 150 ng genomic DNA was spiked with 1.5 pg or 0.15 pg (0.15 pg/150 ng approximates 1 provirus copy per cell) plasmid DNA containing the XMRV *env* sequence (provided by F. Ruscetti). Nested PCR was carried out on 1.5 µl of the first round product in a 25 μl reaction as previously described for *gag* [94] and at an annealing temperature of 58°C using primers 5942F (5′-GGGGACGATGACAGACACTTTCC) and 6271R (5′-GAGCCCACTGAGGAATCAAAACAG) for *env*. PCR products were analyzed by electrophoresis on 1.5-2% agarose gels and staining with ethidium bromide. 

Nested PCR was carried out as described [62] to test for the presence of mouse mitochondrial DNA in 250 ng EKVX genomic DNA. Single-round PCR for intracisternal A particles (IAP) for 45 cycles on up to 600 ng of EKVX genomic DNA was carried out as published [63]. 

Additional PCR products were generated for sequencing using Finnzyme Phusion polymerase (New England Biolabs, Ipswich, MA) with up to 200 ng genomic DNA in 100 µl reactions containing the HF buffer supplied by the manufacturer, 200 μM dNTPs, and 0.5 μM each primer. Primers were designed to amplify regions in the LTR, *gag*, and *env* based on the sequence of DG-75 X-MLV (GenBank accession no. AF221065). Primers and conditions used were: DG-75 7762F (5′-CAAGCTAGCTGCAGTAACGCCATT) and DG-75 287R (5′-CAGACGCAGGCGCAAACATTAGAT) with initial denaturation for 30 s at 98°C followed by 35 cycles of denaturation at 98°C for 8 s and annealing/extension at 72°C for 15 s, followed by a final extension at 72°C for 7 min; DG-75 7607F (5′-AAAGGCAGAATTTCGGTGGTGCAG) and DG-75 7968R (5′-TGCTGGTTCCGCTTTATCTGGGTA) with the same conditions; and DG-75 7945R (5′-TACCCAGATAAAGCGGAACCAGCA) and DG-75 664R (5′-AGGGTCAGACTCAAAGGAGTGGTT) with initial denaturation for 30 s at 98°C followed by 35 cycles of denaturation at 98°C for 8 s, annealing at 70°C for 20 s, and extension at 72°C for 26 s, followed by a final extension at 72°C for 7 min. Products were separated on 2% agarose gels and extracted using a QiaexII Gel Extraction Kit (Qiagen, Valencia, CA). 

### 3.4. Sequencing and Alignment

Sanger sequencing was performed by the Laboratory of Molecular Technology, SAIC-Frederick. Sequences were assembled using Geneious v5.1.6 [95]. Sequences were aligned with representative XMRV and X-MLV sequences using Clustal W2 (www.ebi.ac.uk/Tools/msa/clustalw2) [96,97]. Sequences have been deposited with GenBank with accession numbers JN861040, JN861041, and JN861042. 

### 3.5. Cell-free Virus Transmission Assay

Supernatants were collected from cultures of EKVX cells 24 hours after a media change and were passed through 0.45 μm filters. For virus infection, subconfluent LNCaP cells in 6-well dishes were pretreated with polybrene (4 μg/ml), then the media was removed and replaced with cell-free EKVX supernatant or fresh media (negative control) containing polybrene (2 μg/ml). After 5 hours incubation, the media was changed in each well. Cells were subcultured and total protein lysates were prepared at 10 and 16 days post-infection. Analysis for viral proteins by immunoblotting was carried out. 

## 4. Conclusions

The human lung adenocarcinoma cell line EKVX produces an X-MLV that is infectious to human cells. Because the EKVX cell line was established following the passage of the original tumor cells in nude mice, the source of the virus may be the activation of an endogenous virus in the mouse rather than the original human tumor. Regardless of the origin of the X-MLV in the cell line EKVX, its discovery serves as a reminder to handle all human-derived cell lines, even those tested for known human pathogens, with caution. 
